# Prevalence and associated factors of alexithymia among people living with HIV/AIDS in China: a cross-sectional study

**DOI:** 10.1186/s12888-023-04932-4

**Published:** 2023-06-13

**Authors:** Huan Liu, Yisi Yang, Yingnan Tian, Shanshan Gao, Yunxia Ma, Yuxuan Wang, Ling Xin, Nana Luo, Xinyu Wang, Nan Meng, Ruiqian Zhuge, Qunkai Wang, Qunhong Wu, Baohua Liu

**Affiliations:** 1 Department of Health Policy, School of Health Management, Harbin Medical University, Harbin, China; 2 Department of Social Medicine, School of Public Health, Harbin Medical University, Harbin, China; 3 Harbin Center for Disease Control and Prevention, Harbin, , China; 4 School of Business and Economics, University of San Carlos, Cebu, Philippines; 5 School of Health Service and Healthy Elderly Care, Ningbo College of Health Sciences, Ningbo, Zhejiang China

**Keywords:** Alexithymia, Antiretroviral therapy side effects, Financial burden, Loneliness

## Abstract

**Background:**

Alexithymia is common and causes serious harm to people living with HIV/AIDS. Therefore, this study aimed to examine its prevalence and associated factors among people living with HIV/AIDS in China.

**Methods:**

A cross-sectional study was conducted in two designated AIDS medical institutions in Harbin, China between January and December 2019. In total, 767 participants completed the 20-item Toronto Alexithymia Scale, the University of California Los Angeles Loneliness short-form, the Patient Health Questionnaire-9, the HIV Treatment Regimen Fatigue Scale, and the Alcohol Use Disorders Identification Test‐Consumption. The participants responded to several questions regarding their demographic characteristics, life satisfaction, disease-related economic burden, and their antiretroviral therapy (ART) side effects. Multivariate logistic regression assessed the relationship between alexithymia and associated factors. Odds ratios (OR) and 95% confidence intervals (CI) for OR were calculated.

**Results:**

Approximately 36.1% of the participants were classified as having alexithymia. After adjusted age and education, the logistic regression model indicated that disease-related economic burden (OR = 1.477, 95% CI = 1.155–1.888), ART side effects (OR = 1.249, 95% CI = 1.001–1.559), loneliness (OR = 1.166, 95% CI = 1.101–1.236), and HIV treatment regimen fatigue (OR = 1.028, 95% CI = 1.017–1.039) were positively associated with alexithymia.

**Conclusions:**

The mental health problems of people living with HIV/AIDS are essential to understand and deserve attention. Disease-related economic burdens are major associated factors. Multiple actors should provide better services and guarantees for patients.

## Background

In the 1960s and 1970s, Sifneos, a psychotherapist, first proposed the word alexithymia [1] to describe a personality trait rather than a disorder [2]. Alexithymia refers to difficulty in identifying and distinguishing between feelings and bodily sensations, difficulty in describing feelings, restricted imaginal capacity, and externally oriented thinking [1, 3]. Individuals with alexithymia have a poor ability to process and regulate emotions, and hence, it is also known as "emotional inability" or "emotional dyslexia." Some studies suggest that alexithymia is a risk factor for various mental or physical diseases [4–6]. Alexithymia was first widely reported among individuals with psychosomatic diseases [1], and then extended to other groups including university students, prisoners [7, 8], and the patient population [9, 10]. Some studies have shown that the prevalence of alexithymia is higher in clinical populations than in the general population [11, 12]. However, no empirical study has clarified the prevalence of alexithymia among people living with HIV/AIDS.

AIDS, caused by HIV, remains a global crisis. According to the Joint United Nations Programme on HIV/AIDS, 38.4 million PLWHA and 650,000 people died of AIDS-related diseases in 2021 [13]. Compared with other countries, China's HIV/AIDS situation is the most prominent, both in terms of an increasing trend of infected people and a high mortality rate [7, 14]. PLWHA have a heavy psychological burden and are at risk of developing mental illnesses, including depression and loneliness, as a result of stigma, discrimination, and social isolation, which leads to poorer health outcomes and quality of life among them. Several pieces of evidence indicate that mental health problems may also limit people's emotional experience and aggravate the severity of alexithymia [15–17] and their general condition. However, alexithymia in HIV/AIDS patients has been inadequately studied [2], and factors associated with alexithymia in PLWHA are scarce.

The underlying socio-demographic factors associated with alexithymia in different populations may include age, education [8], marital status, and socioeconomic status [18]. In addition, according to the secondary alexithymia theory, alexithymia can be interpreted as a protective strategy. People with negative affect typically engage in emotional inhibition strategies to cope with their symptoms [19], and thus experience more difficulty with subjectively identifying and describing their emotions [20]. In other words, people with negative affect are most likely to have a high level of alexithymia. Furthermore, as a defense or strategy for coping with emotional distress, higher alcohol use disorder has been associated with alexithymia, and alcohol may play a role in social coping strategies [18]. Some scholars suggested that alcohol can alleviate tension and improve interpersonal performance in individuals with alexithymia, which may increase the possibility of alcohol abuse among this population [15]. However, compared with the general population, alcohol consumption causes greater damage to the health of HIV-infected patients [21].

Disease status and treatment-related factors of PLWHA also deserve more attention. Previous studies revealed that alexithymia may be related to HIV disease severity and adherence to antiretroviral therapy (ART) [22]. The common factors related to the treatment adherence of PLWHA include ART side effects [23] and treatment regimen fatigue [24]. “Four Frees and One Care” policy, which is one of the most important recent policies from the Chinese Government to fight against the HIV/AIDS pandemic, has substantially reduced the economic burden of HIV diagnosis and treatment on patients in China. However, compared to the general population, HIV-infected individuals have a higher risk of complications, including cardiovascular disease and cancer [25]. These complications undoubtedly cause certain economic burden to the PLWHA; however, the relationship between disease-related economic burden and alexithymia has yet to be explored in PLWHA.

Consequently, this study aimed to investigate: (1) the percentage of different alexithymia severities in AIDS patients, and (2) the potential associated factors of alexithymia.

## Method

### Data collection and population study

Harbin, the capital city of Heilongjiang province in Northeast China, has a population of approximately 10 million people, posing significant challenges to AIDS prevention and control [26]. A cross-sectional survey of PLWHA who received ART was conducted in the fourth affiliated hospital of Harbin Medical University and the city’s sixth hospital (Harbin Infectious Disease Hospital) between January and December 2019. Patients were selected based on the following criteria: aged 18 years or older, PLWHA, a receiver of ART, and the ability to read and write in Chinese. Patients who met the criteria were informed by their physicians about the study and provided consent to participate. All eligible participants were informed that participation was voluntary and that they could withdraw at any time. Confidentiality was ensured as the study used identification numbers to protect the privacy of participants. After obtaining their consent by signing the informed consent form, specially-trained researchers conducted the survey. Ethics approval was obtained from the ethics review committee of the Harbin Center for Disease Control and Prevention. In total, 1,115 questionnaires were completed after eliminating the samples with more than half of the questions with incomplete answers and those answered incorrectly. The respondents who did not completely answer the alexithymia questions were eliminated. Ultimately, 767 questionnaires (69.1%) were deemed valid and used for data analysis.

### Variables

A structured questionnaire was designed to obtain data regarding demographics and social-economic characteristics, alexithymia, loneliness, depression, treatment regimen fatigue, hazardous alcohol consumption, life satisfaction, ART side effects, and disease-related economic burden.

### Dependent variable

The dependent (outcome) variable was alexithymia. It was measured using the Chinese version [27] of the 20-item Toronto Alexithymia Scale (TAS-20) [28]. Each of these 20 items is rated on a five-point Likert scale (1 = “strongly disagree” to 5 = “strongly agree”). According to the original classification criteria, participants who scored ≤ 51 were classified as non-alexithymia, those who scored between 52 and 60 were classified as possibly having alexithymia, and those who scored 61 or above were classified as alexithymia [28]. For study purposes, participants were classified as alexithymia or non-alexithymia (non-alexithymia and possibly having alexithymia). This classification has been previously used by other authors [29, 30]. In this study, the TAS-20 showed acceptable internal consistency (Cronbach’s α = 0.739, McDonald’s omega = 0.748).

### Independent variable

Loneliness was measured using the Chinese version [31] of the University of California Los Angeles Loneliness Scale-6 (ULS-6) [32]. It comprises six items rated by the participants, of which five items (e.g., “How often do you feel isolated from others?”) were worded negatively and one item worded positively [32]. The participants answered the items on a 4-point scale ranging from 1 (never) to 4 (often) with a total score between 6 and 24, in which a higher score indicates greater loneliness. In this study, the UlS-6 exhibited a high level of internal consistency (Cronbach’s α = 0.834, McDonald’s omega = 0.836).

Depression was assessed using the Patient Health Questionnaire-9 (PHQ-9). It included nine items (e.g., “little interest or pleasure in doing things”), and each item was scored from 0 (not at all) to 3 (nearly every day). As a result, the PHQ-9 total score ranges from 0 to 27. According to the recommendation of Kroenke et al., a PHQ-9 score > 9 was used to identify participants with a possible depressive disorder [33]. The PHQ-9 has shown good reliability and validity in previous studies of HIV-infected individuals in China [34, 35]. The McDonald’s omega of this scale was 0.850 and Cronbach’s α was 0.849, indicating that it has good reliability.

Treatment regimen fatigue was measured using the HIV Treatment Regimen Fatigue Scale developed by Claborn et al. [36]. The scale is a 22-item measure of mental fatigue, cynicism, and self-efficacy to adhere to treatment [36]. Responses for each item range from − 3 (strongly disagree) to + 3 (strongly agree), with higher total scores indicating higher levels of treatment regimen fatigue. The HIV Treatment Regimen Fatigue Scale showed good reliability (Cronbach’s α = 0.866, McDonald’s omega = 0.851).

The level of alcohol use was measured using the Alcohol Use Disorders Identification Test-Consumption (AUDIT-C) [37]. Participants were invited to answer, “How often did you have a drink containing alcohol in the past year?” with the following five response options: never (0 points); monthly or less (1 point), two to four times a month (2 points), two to three times a week (3 points), four to five times a week (4 points), or six or more times a week (4 points). Participants were asked, “How many drinks did you have on a typical day when you were drinking in the past year?” The response categories were 0 = drinks; 1 = 1 to 2 drinks, 2 = 3 to 4 drinks, 3 = 5 to 6 drinks, and 4 = 7 to 9 drinks (3 points). Moreover, participants were invited to report the frequency of drinking six or more drinks on one occasion in the past year. Response options were never (0 points); less than monthly (1 point); monthly (2 points); weekly (3 points); or daily or almost daily (4 points). Those with a total score of three or more indicated heavy drinking and/or active alcohol abuse or dependence [37]. Cronbach’s α and McDonald’s omega of this scale were 0.927 and 0.948, respectively, indicating high internal consistency.

Moreover, life satisfaction was measured with the following question: “Are you satisfied with your life at present?” Participants were asked to choose 1 answer for the statement (1 = very dissatisfied, 2 = dissatisfied, 3 = neutral (neither satisfied nor dissatisfied), 4 = satisfied, and 5 = very satisfied). ART side effects were measured using the question, “How much do the side effects of ART affect you?” The response categories were 1 = rarely or not at all, 2 = little, 3 = average, 4 = significantly, 5 = severely. Disease-related economic burden was measured with the question, “What is the current level of your financial burden for the treatment of HIV and related illnesses?” The participants’ responses were rated on a 5-point Likert scale (1 = almost none, 2 = less, 3 = medium, 4 = heavy, and 5 = very heavy).

Demographic and socio-economic characteristics included age, sex, marital status, educational background, household monthly income per capita, disease duration, infection route, CD4 + cell count, and viral load*.* The coding of all categorical independent variables is shown in Table 1.

**Table 1:** Categorical independent variables and coding

Variable	Characteristic	Coding
**Age (year)**		
	≤ 30	1
	31 ~ 35	2
	36 ~ 41	3
	42 ≥	4
**Sex**		
	Male	1
	Female	0
**Marital status**		
	Married or cohabitating	1
	Unmarried, divorced, or widowed	0
**Education background**		
	Junior high school or below	1
	High school/secondary school	2
	College, undergraduate or above	3
**Household monthly income per capita**		
	3000 yuan or below	1
	3000 to < 5000 yuan	2
	5000 yuan or above	3
**Location**		
	Urban	1
	Rural	0
**Disease-related economic burden**		
	Almost none	1
	Less	2
	Medium	3
	Heavy	4
	Very heavy	5
**Alcohol abuse**		
	Yes	1
	No	0
**CD4 + cell numbers (cells/μL)**		
	< 200	1
	200–499	2
	≥ 500	3
**Viral load (copies/ml)**		
	< 50	0
	≥ 50	1
**ART side effects**		
	rarely or not at all	1
	little	2
	average	3
	significantly	4
	severely	5
**Life satisfaction**		
	Very dissatisfied	1
	Dissatisfied	2
	Neutral	3
	Satisfied	4
	Very satisfied	5
**Depression**		
	Yes	1
	No	0

### Statistical analyses

We used the Q-Q plot, skewness, and kurtosis tests to comprehensively examine the distribution normality for each continuous variable. According to Kim, for samples > 300, the approximate normal distribution was defined for variables with absolute values of skewness below 3 and kurtosis below 8 [38]. The results showed that the skewness values of each continuous variable ranged from -1.51 to 0.95 and the kurtosis values ranged from -0.82 to 2.11, indicative of normal distribution [38] (for details, see Table 2). The Q-Q plot test result indicated that all the continuous variables did not seriously deviate from the normal distribution except for the variable of HIV Treatment Regimen Fatigue.

**Table 2:** Mean, standard deviation, skewness, and kurtosis of variables

Variables	Total				Alexithymia				Non- Alexithymia			
	Mean	SD	Skewness	Kurtosis	Mean	SD	Skewness	Kurtosis	Mean	SD	Skewness	Kurtosis
TAS-20	57.21	9.14	-0.66	0.97	66.05	4.15	0.95	0.31	52.21	7.21	-1.51	2.11
UlS-6	12.31	3.94	0.31	-0.17	14.13	4.20	0.03	-0.34	11.29	3.37	0.18	-0.39
HIV Treatment Regimen Fatigue Scale	-15.84	22.59	-0.69	-0.52	-8.99	20.35	-0.72	-0.20	-19.72	22.90	-0.66	-0.82

Descriptive statistics were analyzed using frequencies and percentages for binary/categorical variables, means, and standard deviations (SD) for normally distributed continuous variables. Median and interquartile ranges were presented for continuous variables that were not normally distributed. Univariate analysis was performed using Pearson’s chi-squared test, t-tests, Wilcoxon’s rank sum test, Pearson correlation analysis, and Spearman correlation analysis. Subsequently, the independent variables presenting *P* value < 0.05 were included in the unadjusted model (Model 1). Meanwhile, we also further adjusted for age and education in Model 2. Multivariate logistic regression was conducted to examine associations between alexithymia and associated factors. Odds ratios (OR) and 95% confidence intervals (CI) for OR were calculated. All data were analyzed using SPSS 26.0, and *P* < 0.05 was considered significant.

## Results

### Participants’ characteristics

As shown in Table 3, participants were divided into three groups based on the severity of alexithymia. In the total sample (*N* = 767), alexithymia accounted for approximately 36.1%. We analyzed the general characteristics of respondents with alexithymia at different levels. Within the total sample (*N* = 767), most were male (96.3%); unmarried, divorced, or widowed (78.2%); were undergraduate or had higher education level (39.0%); household monthly income ranged from 3000 to 5000 yuan (47.1%) and majority participants live in urban areas (73.4%). Univariate analysis results show that educational background, household monthly income, disease-related economic burden, location, CD4 + cell numbers, ART side effects, life satisfaction, depression, HIV treatment regimen fatigue, and loneliness reflect statistical differences in different groups (*P* < 0.001).

**Table 3:** Participants’ characteristics based on their alexithymia status

Variables	Total (*N* = 767)	Alexithymia (*n* = 277)	Non- Alexithymia (*n* = 490)	*P*-value^d^
**Age (year)** ^**a**^				0.491
≤ 30	186 (24.3)	62 (22.4)	124 (25.3)	
31 ~ 35	185 (24.1)	63 (22.7)	122 (24.9)	
36 ~ 41	199 (25.9)	80 (29.0)	119 (24.3)	
42 ≥	191 (24.9)	68 (24.5)	123 (25.1)	
Missing data	6 (0.8)	4 (1.4)	2 (0.4)	
**Sex** ^**a**^				0.316
Male	739 (96.3)	264 (95.3)	475 (96.9)	
Female	28 (3.7)	13 (4.7)	15 (3.1)	
**Marital status** ^**a**^				0.651
Married or cohabitating	99 (12.9)	37 (13.4)	62 (12.7)	
Unmarried, divorced, or widowed	600 (78.2)	210 (75.8)	390 (79.6)	
Missing data	68 (8.9)	30 (10.8)	38 (7.7)	
**Education background** ^**a**^				0.435
Junior high school or below	142 (18.5)	54 (19.5)	88 (18.0)	
High school/secondary school	286 (37.3)	104 (37.6)	182 (37.1)	
College, undergraduate or above	299 (39.0)	97 (35.0)	202 (41.2)	
Missing data	40 (5.2)	22 (7.9)	18 (3.7)	
**Household monthly income per capita** ^**a**^				0.008
3000 yuan or below	207(27.0)	93 (33.6)	114 (23.3)	
3000 to < 5000 yuan	361(47.0)	117 (42.2)	244 (49.8)	
5000 yuan or above	193 (25.2)	65 (23.5)	128 (26.1)	
Missing data	6 (0.8)	2 (0.7)	4 (0.8)	
**Location** ^**a**^				0.068
Urban	428 (55.9)	142 (51.3)	286 (58.4)	
Rural	288 (37.5)	155 (41.5)	173 (35.3)	
Missing data	51 (6.6)	20 (7.2)	31 (6.3)	
**Disease-related economic burden** ^**a**^				< 0.001
Almost none	293 (38.2)	84 (30.3)	209 (42.6)	
Less	211 (27.5)	75 (27.1)	136 (27.8)	
Medium	181 (23.6)	69 (24.9)	112 (22.9)	
Heavy	55 (7.2)	31 (11.2)	24 (4.9)	
Very heavy	24 (3.1)	18 (6.5)	6 (1.2)	
Missing data	3 (0.4)	0 (0.0)	3 (0.6)	
**Alcohol abuse** ^**a**^				0.811
Yes	262 (34.2)	92 (33.2)	170 (34.7)	
No	497 (64.8)	180 (65.0)	317 (64.7)	
Missing data	8 (1.0)	5 (1.8)	3 (0.6)	
**CD4 + cell numbers (cells/μL)** ^**a**^				0.006
< 200	40 (5.2)	12 (4.3)	28 (5.7)	
200–499	212 (27.6)	95 (34.3)	117 (23.9)	
≥ 500	505 (65.9)	165 (59.6)	340 (69.4)	
Missing data	10 (1.3)	5 (1.8)	5 (1.0)	
**Viral load (copies/ml)** ^**a**^				0.047
< 50	674 (87.8)	250 (90.3)	424 (86.6)	
≥ 50	78 (10.2)	20 (7.2)	58 (11.8)	
Missing data	15 (2.0)	7 (2.5)	8 (1.6)	
**ART side effects** ^**a**^				< 0.001
Rarely or not at all	304 (39.6)	83 (30.0)	221 (45.1)	
Little	158 (20.6)	49 (17.7)	109 (22.2)	
Average	237 (30.9)	100 (36.1)	137 (28.0)	
Significantly	52 (6.8)	35 (12.6)	17 (3.5)	
Severely	13 (1.7)	10 (3.6)	3 (0.6)	
Missing data	3 (0.4)	0 (0)	3 (0.6)	
**Life satisfaction** ^**a**^				0.044
Very dissatisfied	44(5.7)	21 (7.6)	23 (4.7)	
Dissatisfied	85 (11.1)	40 (14.4)	45 (9.2)	
Neutral	408 (53.2)	137 (49.5)	271 (55.3)	
Satisfied	175 (22.8)	56 (20.2)	119 (24.3)	
Very satisfied	36 (4.7)	11 (4.0)	25 (5.1)	
Missing data	19 (2.5)	12 (4.3)	7 (1.4)	
**Depression** ^**a**^				< 0.001
Yes	390 (50.8)	169 (61.0)	221 (45.1)	
No	377 (49.2)	108 (39.0)	269 (54.9)	
**Loneliness ** ^**b**^	12.31 ± 3.94	14.13 ± 4.20	11.29 ± 3.37	< 0.001
**HIV treatment regimen fatigue** ^**c**^	-10 (-31–0)	-5 (-22–8)	-12 (-38–-2)	< 0.001

^a^Data were expressed as numbers. (%); ^b^Data are presented as mean ± standard deviation; ^c^Data were expressed as median (P25–P75); ^d^*P*-value was calculated using the chi-square test, t-test, and Wilcoxon’s rank-sum test where applicable

### Score analysis of TAS-20

**Fig. 1:**
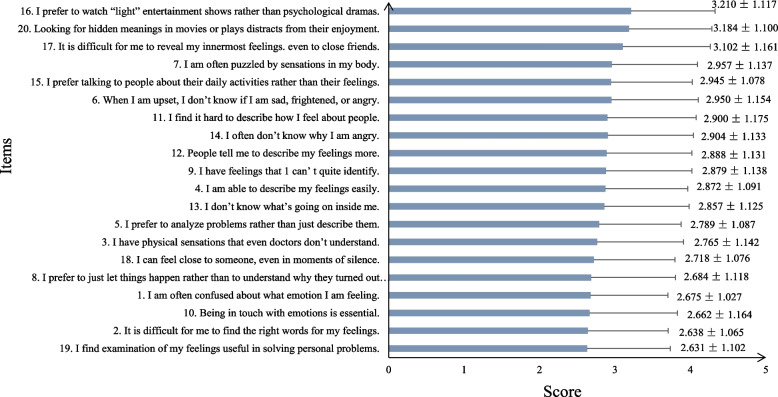
TAS-20 score for each item

The mean (SD) score of participants’ TAS-20 was 57.21(9.14). Figure 1 shows the means and standard deviations of each item in TAS-20. Sort based on the mean score of each item, “I prefer to watch ‘light’ entertainment shows rather than psychological dramas” is a major manifestation of alexithymia (3.210 ± 1.117; mean ± SD), followed by “Looking for hidden meanings in movies or plays distracts from their enjoyment” (3.184 ± 1.100; mean ± SD). In addition, “It is difficult for me to reveal my innermost feelings, even to close friends” (3.102 ± 1.161; mean ± SD), “I am often puzzled by sensations in my body” (2.957 ± 1.137; mean ± SD), “I prefer talking to people about their daily activities rather than their feelings” (2.945 ± 1.078; mean ± SD) also were key manifestations of alexithymia, respectively.

### Logistic regression analysis of factors associated with alexithymia

Independent variables that were significant predictors in the univariate analysis were entered into the logistic regression analysis model. We found that disease-related economic burden (OR = 1.439, 95% CI = 1.143–1.813), ART side effects (OR = 1.251, 95% CI = 1.014–1.543), loneliness (OR = 1.174, 95% CI = 1.100–1.242), and HIV treatment regimen fatigue (OR = 1.027, 95% CI = 1.017–1.037) were important risk factors for alexithymia in PLWHA in the Model 1 (Table 4). After adjustment for age and education background, we found that disease-related economic burden (OR = 1.477, 95% CI = 1.155–1.888), ART side effects (OR = 1.249, 95% CI = 1.001–1.559), loneliness (OR = 1.166, 95% CI = 1.101–1.236), and HIV treatment regimen fatigue (OR = 1.028, 95% CI = 1.017–1.039) were important risk factors for alexithymia in PLWHA in the Model 2 (Table 4).

**Table 4:** Logistic regression analysis of factors associated with alexithymia

Variables	*Model 1*					*Model 2*				
	*β*	*Standard error*	*Wald*	P	OR (95% CI)	*β*	*Standard error*	*Wald*	P	OR (95% CI)
Household monthly income per capita	-0.011	0.129	0.007	0.935	0.989 *(0.768*–*1.275)*	0.049	0.136	0.129	0.720	1.050 (0.805–1.370)
Disease-related economic burden	0.364	0.118	9.596	0.002	1.439 *(1.143*–*1.813)*	0.390	0.125	9.664	0.002	1.477(1.155–1.888)
CD4 + cell numbers (cells/μL)	-0.142	0.151	0.892	0.345	0.867 *(0.645*–*1.165)*	-0.105	0.155	0.457	0.499	0.901(.665–1.220)
Viral load (copies/ml)	-0.408	0.306	1.772	0.183	0.665 (*0.365*–1.212)	-0.414	0.314	1.745	0.186	0.661(0.357–1.222)
ART side effects	0.224	0.107	4.382	0.036	1.251 *(1.014*–*1.543)*	0.223	0.113	3.880	0.049	1.249(1.001–1.559)
Life satisfaction	0.225	0.116	3.755	0.053	1.252 *(0.997*–*1.571)*	0.187	0.121	2.396	0.122	1.205(0.951–1.527)
Depression	0.100	0.107	0.866	0.352	1.105 *(0.895*–*1.364)*	0.124	0.111	1.248	0.264	1.131(0.911–1.405)
Loneliness	0.161	0.029	31.677	< 0.001	1.174 *(1.100*–*1.242)*	0.154	0.030	27.172	< 0.001	1.166(1.101–1.236)
HIV treatment regimen fatigue	0.026	0.005	26.317	< 0.001	1.027 *(1.017*–*1.037)*	0.027	0.005	24.802	< 0.001	1.028(1.017–1.039)

Model 1: unadjusted; Model 2 adjusted for age and education

## Discussion

In this study, the prevalence of alexithymia in PLWHA was approximately 36.1%. The disease-related economic burden is the most important associated factor for alexithymia in PLWHA. The results can be utilized by policymakers to relieve alexithymia in PLWHA.

The disease-related economic burden is associated with the occurrence of alexithymia, especially in PLWHA, which is a long-term disease [39]. First, China introduced policies on remission and assistance to reduce the financial burden of PLWHA to a certain extent. Nevertheless, the cost of long-term care for patients remains high due to the side effects of antiretroviral drugs, recurrent co-infections, and chronic illnesses that occur with age [40, 41]. A cohort study found that approximately 70% of PLWHA people have at least one or more chronic non-communicable comorbidities, which is 1.12 times higher than the general population. Additionally, it has been shown that the more chronic diseases, the higher the level of alexithymia [42]. Second, PLWHA patients have limited work opportunities because of their poor physical condition, failed medical examinations for employment, and other factors. Furthermore, the high financial burden associated with the disease may increase the psychological stress of patients and the possibility of alexithymia [43]. Moreover, some PLWHA may give up on treatment for themselves and sell their free antiretroviral drugs to earn an income, which may deteriorate their condition and make them threatening to society [18]. Several patients do not meet their needs because of the limited number of free drugs. Therefore, providing more quality drugs in health insurance or making them free will effectively reduce the disease-related economic burden for PLWHA. Moreover, while the government should strictly control the distribution of free drugs, it should also appropriately relax restrictions on the employment of PLWHA. Communities could offer job training to help PLWHA find employment or provide opportunities.

PLWHA must receive lifelong treatment. Our study found that the side effects of long-term ART may be associated with alexithymia in HIV patients, and this lifelong treatment is also an endurance test for patients who are highly vulnerable to treatment regimen fatigue which may further exacerbate their risk of acquiring alexithymia. This is consistent with previous findings [44, 45]. However, PLWHA face multiple pressures of chronic fear of disease disclosure, have limited psychosocial support, and lower socioeconomic status. Treatment side effects are highly susceptible to treatment regimen fatigue and may indirectly precipitate other psychological problems, such as the development of anxiety and depressive symptoms that further exacerbate alexithymia [7, 40, 46, 47]. Thus, pharmacological neuroprotective therapies can be combined with low-cost, effective treatments such as cognitive therapy and exercise to complement free ART to mitigate the effects of its long-term side effects on alexithymia in PLWHA [46]. Moreover, most notably, the long-standing "Four Frees and One Care" policy, implemented in 2003 for PLWHA in China, has consistently neglected their psychological care [42], and hence, there is an urgent need for the government to provide fully free psychological counseling and outpatient services for HIV patients to help prevent and manage mental illness.

Our study found that loneliness was associated with alexithymia. HIV patients' poor health status reduces their communication with the outside world, and the lack of psychological support, facilitation, and disease exposure can lead to increased cognitive load and reduced physical executive functioning, which makes it difficult to describe feelings to others [48]. From the lack of daily communication with others, HIV/AIDS patients who experience loneliness for a long period may develop a metabolic imbalance in their bodies that causes an inability to distinguish between somatic arousal sensations and internal feelings, a tendency to replace mental emotions with somatic sensations, with a reduced ability to detect and recognize emotions in themselves and the outside world. Therefore, caring for PLWHA requires social cooperation, such as with family, community, and medical institutions. Moreover, all provinces should establish social organization service centers for PLWHA to conduct publicity work and provide care and companionship for patients.

Despite the strength of this study, some limitations need to be mentioned. First, as this study was cross-sectional, it was unable to establish causality. Second, the analysis was not pre-registered, and the results should therefore be considered exploratory [49]. Third, information on the exposure and outcome was obtained through a self-reported questionnaire that potentially constituted a reporting bias; however, it would most likely be a non-differential bias. Fourth, the sample size is small and cannot be considered representative. Therefore, caution is recommended when generalizing the results. In the future, a larger scope of investigations and sample size are required to reveal whether interventions from different actors can effectively reduce alexithymia in PLWHA patients.

## Conclusion

Approximately 36.1% of the respondents have alexithymia. The major finding was that PLWHA, ART side effects, disease-related economic burden, loneliness, and HIV treatment regimen fatigue were positively associated with alexithymia. Currently, mental health issues in PLWHA are not being effectively addressed. Medical institutions should focus on the side effects of the treatment process. Furthermore, the government should consider providing completely free psychological counseling services for PLWHA, and comprehensive protection against patients' disease-related economic burdens. Communities should assume greater responsibilities, including providing employment opportunities, publicity, and education for PLWHA. The findings are expected to provide references for clinicians to prevent and treat alexithymia in PLWHA, and guide policymakers to improve AIDS-related policies, further relieving the mental health problems of PLWHA. Future research should explore the impact of different interventions on alexithymia among PLWHA.

## Data Availability

On the one hand, the data involves the use of subsequent research, and on the other hand, the research involves sensitive populations. Therefore, the data in this study cannot be shared.

## Abbreviations

AUDIT-C: Alcohol Use Disorders Identification Test –consumption
ART: Antiretroviral therapy
CI: Confidence interval
OR: Odd ratio
PHQ-9: Patient Health Questionnaire-9
PLWHA: People living with HIV/AIDS
SD: Standard deviations
TAS-20: 20-Item Toronto Alexithymia Scale
UlS-6: University of California Los Angeles Loneliness Scale-6
